# Mass social media-induced illness presenting with Tourette-like behavior

**DOI:** 10.3389/fpsyt.2022.963769

**Published:** 2022-09-20

**Authors:** Carolin Fremer, Natalia Szejko, Anna Pisarenko, Martina Haas, Luise Laudenbach, Claudia Wegener, Kirsten R. Müller-Vahl

**Affiliations:** ^1^Clinic of Psychiatry, Social Psychiatry and Psychotherapy, Hannover Medical School, Hanover, Germany; ^2^Department of Neurology, Medical University of Warsaw, Warsaw, Poland; ^3^Department of Bioethics, Medical University of Warsaw, Warsaw, Poland; ^4^Department of Audiovisual Media Studies, Film University Babelsberg, Potsdam, Germany

**Keywords:** Tourette-like behavior, social media, mass sociogenic illness, functional movement disorders, mass social media-induced illness

## Abstract

Currently, we are facing a new manifestation of functional neurological disorder presenting with functional Tourette-like behavior (FTB). This study aimed to show characteristics of this phenotype presenting as an outbreak of “mass social media-induced illness” (MSMI) and to explore predisposing factors. Between 5–9/2021, we prospectively investigated 32 patients (mean/median age: 20.1/18 years, range: 11–53 years, *n* = 16 females) with MSMI-FTB using a neuro-psychiatric examination, a comprehensive semi-structured interview and aspects of the Operationalized Psychodynamic Diagnostic System. In contrast to tics, numbers of complex movements and vocalizations were nine times greater than of “simple” symptoms, and of vocalizations one and a half times greater than of movements. In line with our hypothesis of MSMI, symptoms largely overlapped with those presented by German YouTuber Jan Zimmermann justifying his role as “virtual” index case in current outbreak. Typically, symptoms started abruptly at a mean age of 19 years and deteriorated gradually with no differences between males and females. In all patients, we identified timely-related psychological stressors, unconscious intrapsychic conflicts, and/or structural deficits. Nearly all patients (94%) suffered from further psychiatric symptoms including abnormalities in social behavior (81%), obsessive-compulsive behavior (OCB) (47%), Tourette syndrome (TS) (47%), anxiety (41%), and depression (31%), about half (47%) had experienced bullying, and 75% suffered from coexisting somatic diseases. Our data suggest that pre-existing abnormalities in social behavior and psychiatric symptoms (OCB, anxiety, and depression), but also TS in combination with timely-related psychological stressors, unconscious intrapsychic conflicts, and structural deficits predispose to contagion with MSMI-FTB.

## Introduction

Only recently, our group suggested the concept of a new type of mass sociogenic illness (MSI) spread solely *via* social media (1). So far, it was believed that outbreaks of MSI in any case require *direct* face-to-face contact among persons affected (2). In Germany, we now identified an outbreak of functional Tourette-like behavior (FTB) spread *via* social media and suggested the more specific term “mass social media-induced illness” (MSMI) (1). Functional tic-like symptoms have been considered a relatively rare manifestation of functional neurological disorders (FND) accounting for only 2–5 % of all FND (3, 4). However, currently, a tremendous increase in FTB has been noticed (1, 5–12) with a remarkable clinical overlap including teenage age, female preponderance, relatively abrupt onset of mainly complex tic-like symptoms and socially inappropriate behaviors. Even kind of symptoms show an astonishing similarity with complex, stereotyped movements at arms and body and vocalizations dominated by swear words and insults (1, 5–12). In line with older reports (13, 14), in a small number of patients, preexisting tics/Tourette syndrome (TS) have been described (6, 11).

Several authors speculated that current increase in FTB is related to the COVID-19 pandemic, increased social media use, and/or “tic-like” presentations on social media (1, 5–12). The latter earns enormous attention among young people with millions of subscribers and followers of the most popular influencers such as Jan Zimmermann with his German YouTube channel “Gewitter im Kopf” (“thunderstorm in the brain”) (15)^1^, the English speaking Evie Meg under her TikTok name “thistrippyhippie” (16, 17)^2^^,^ ^3^, and the Danish speaking Stine Sara (18)^4^ Interestingly, not only patients affected, but also these influencers show a remarkable symptom overlap.

As outlined recently (1), in Germany we suggested that the YouTuber Jan Zimmermann acts as a “virtual” index case of an outbreak of MSMI-FTB. In that regard, the following aspects are worth mentioning: in February 2019, the 23-year-old launched the channel “Gewitter im Kopf”. Claiming to inform about TS, his channel reached 1 million subscribers in < 3 months and currently counts 2.21 million subscribers with 315,826,001 views of so far 336 videos released (status 20^th^ Dec 2021) (1, 19)^5^ In his videos (20–27)^6^^–^^13^, he states that he experienced first mild tics before entering elementary school, but by the age of 18, symptoms changed and vocalizations occurred such as whistling and pronunciation of the German words “Lavendel” (“lavender”), “Zuckerwatte” (“cotton candy”), and “Salami” (“salami”) followed by first-ever swear words and whole sentences, pronounced with changed voice with low or high pitch. Stress, presence of police, and people with TS would result in an increase of symptoms. However, most intensive symptoms with most aggressive content would be caused by the presence of his mother. On the contrary, symptoms would completely recede during sleep, intimacy, and sexual intercourse, and when speaking English. After having been diagnosed with TS at age 18, several treatments would have been initiated without improvement. Because of these newly developed symptoms, he stopped his training as a physical therapist and for his driver's license, and as passenger started to sit in the back seat with the child safety lock on. At the same time, he stated that he and his mother found his symptoms “funny” making the family laugh, and his mother took notes of his “funniest phrases”.

Here, we present for the first time detailed clinical data of the largest sample of patients with MSMI-FTB from a single German center described so far. We focussed on the presence of psychological stressors, since they—although removed from diagnostic criteria in DSM-5—are still considered important risk factors for the development of FND reflecting the “conversion” of underlying emotional distress into physical (neurologic) symptoms (28).

## Materials and methods

### Methods

Between 5/2019 and 9/2021, 44 patients attending our Tourette outpatient clinic were diagnosed with FND presenting with Tourette-like symptoms after exposure to the aforementioned social media content. Of these, 32 patients agreed to participate in our study. Firstly, in all patients a thorough neuro-psychiatric examination was carried out by one of the authors (KMV), who is a psychiatrist, neurologist, and TS-expert making the diagnosis of FND and, in addition, confirming or excluding the diagnosis of a concurrent primary tic disorder such as provisional tic disorder or TS according to DSM-5 criteria. Secondly, using an explorative approach by composing a comprehensive semi-structured clinical and psychological interview similar to the one used by Paulus et al. (11), we were able to collect demographic and biographical data, looked for sex differences, and took a detailed history of newly developed symptoms with respect to age and kind of onset, course, triggering factors, suppressibility, distractibility, premonitory sensations, influencing factors, treatment, acceptance of diagnosis of FTB, and coincidence with the pandemic. With the interview composed by the authors and conducted by a psychologist and psychodynamic psychotherapist with extensive experience in TS (CF), we were also able to evaluate underlying psychological mechanisms, triggering and maintaining factors, and pre-existing psychopathology. Depending on patients' age and developmental level, which referred to patient's ability to report in detail, interviews were performed together with the parents. To classify functional movements and vocalizations as “simple” or “complex”, we followed the structure of the Yale Global Tic Severity Scale (YGTSS) (29).

All current and recent movements, vocalizations, and abnormal behaviors reported by patients and parents, and observed during the evaluation were documented. We evaluated use of social media and in particular of the YouTube channel “Gewitter im Kopf” (if parents agreed, without their supervision assuming that this leads to more truthful answers).

Finally, we explored patients with regard to possible underlying emotional distress (e.g., traumatic/stressful life events and family dynamics). Based on the Operationalized Psychodynamic Diagnostic System (30), a widely used and well-established instrument in psychodynamic psychotherapy that aims to explore underlying psychopathology, we evaluated patients intrapsychic conflicts such as conflicting unconscious needs and desires (e.g., dependency and autonomy) and structural deficits, which refer to overall maturity of mental functions and possible lack of certain basic mental abilities (e.g., affect tolerance or regulation of self-esteem), which usually help managing psychological stressors (31).

### Statistical analyses

Data were mainly analyzed descriptively. Frequencies, means, medians, and measures of distribution were calculated using Excel (version Microsoft Office Professional Plus 2016). Analyses for group comparisons were performed in R (version 4.1.1). Within group comparisons, t-tests were performed for interval-scaled dependent variables and Fisher's exact tests for dichotomous dependent variables. *P*-values of significant results for group comparisons were conservatively corrected for multiple comparisons according to Bonferroni (*p*_*c*_). In addition, Cohen's d and bias-corrected Cramer's V were calculated as measures of effect size for significant results.

Detailed text obtained from interviews with respect to psychological and maintaining factors was handled similar to the process of inductive content analysis (32). Accordingly, patients' answers were summarized to categories and, if appropriate, to superordinate categories. Representative answers are provided.

## Results

### General characteristics

Thirty-two patients [mean age: 20.1 years, range: 11–53 years, median age: 18 years, *n* = 16 females (50%)] with MSMI-FTB were included. One male and one female each identified themselves as non-binary. For further analysis, we assigned patients to their biological sex. Interviews were conducted face-to-face (*n* = 22, 68.8%), *via* video (*n* = 9, 28.1%), or phone (*n* = 1, 3.1%). Mean age at onset of MSMI-FTB was 19.2 years (range: 10–53 years, median age: 17 years) with a non-significant difference between males and females (16.8 vs. 21.6 years, *t*_(30)_ = 1.25, *p* = 0.22). For further characteristics see Table 1.

**Table 1:** Clinical characteristics in patients with MSMI-FTB including comparison between males and females.

	**All** ** (*n* = 32)**	**Male** ** (*n* = 16, 50%)**	**Female** ** (*n* = 16, 50%)**	***p* ^a^** ** (m vs. f)**
**Age at evaluation** (years, mean+/-*SD*)	20.1 +/- 11.0	17.8 +/- 9.2	22.4 +/- 12.4	0.234
**Age at onset** (years, mean+/-*SD*)	19.2 +/- 11.0	16.8 +/- 9.0	21.6 +/- 12.5	0.223
**Type of FTB onset** (*n*, %)				
Abrupt Gradual	27 (84.4%) 5 (15.6%)	13 (81.3%) 3 (18.8%)	14 (87.5%) 2 (12.5%)	1 1
**Course of FTB** ^**b**^				
Fluctuations (*n*, %) Slow progression (*n*, %) Improvement (*n*, %)	23 (71.9%) 19 (59.4%) 21 (65.6%)	12 (75%) 8 (50%) 8 (50%)	11 (68.8%) 11 (68.8%) 13 (81.3%)	1 0.473 0.135
**Characteristics of FTB**				
Premonitory sensation (*n*, %) Suppressibility (*n*, %) Distractibility (*n*, %) Rostro-caudal distribution (*n*, %) Temporary remission (*n*, %)	27 (84.4%) 27 (84.4%) 31 (97%) 1 (3%) 25 (78%)	13 (81.3%) 13 (81.3%) 16 (100%) 0 (0%) 12 (75%)	14 (87.5%) 14 (87.5%) 15 (94%) 1 (6%) 13 (81%)	1 0.628 1 1 1
***n*** **of FTB symptoms**				
Simple motor (mean+/-*SD*) Complex motor (mean+/-*SD*) Simple vocal (mean+/-*SD*) Complex vocal (mean+/-*SD*)	1.4 +/- 1.6 11.9 +/- 7.9 2.2 +/- 1.6 18.8 +/- 13.9	0.9 +/- 1.7 12.3 +/- 7.6 2.3 +/- 1.5 21.4 +/- 16.0	1.4 +/- 1.5 11.6 +/- 8.3 2.3 +/- 1.7 16.2 +/- 11.3	0.579 0.792 0.741 0.292

MSMI, Mass social media-induced illness; FTB, functional Tourette-like behavior; SD, standard deviation; m, male; f, female; n, number.

a t-tests were performed for interval-scaled dependent variables and Fisher's exact tests for dichotomous dependent variables.

b Multiple answers possible.

In 19 patients (59.4%) symptoms started before COVID-19 happened in Germany (no information: *n* = 3, 9.4%). Twenty-seven patients (84%) reported an abrupt onset of MSMI-FTB, of whom 25 patients (78.1%) were able to give the exact date (*n* = 6, 18.8%), month (*n* = 10, 31.3%), or time of the year (*n* = 9, 28.1%). Despite (abrupt) onset of new symptoms, seven patients (21.9%) consulted a medical professional for the first time only after 6–12 months. Most patients described a heterogeneous course of FTB with long lasting periods of constant deterioration (*n* = 19, 59.4%), fluctuations (*n* = 23, 71.9%), and symptom improvement (*n* = 21, 65.6%, multiple answers possible).

In none of the patients, the correct diagnosis of MSMI-FND had been made before presentation in our clinic. Instead, in all but two patients (*n* = 30, 93.8%), FND was misdiagnosed as tics/TS. Accordingly, 20 patients (62.5%) had received anti-tic medication (on average, 2.4 different drugs for a mean duration of 4.8 months; for details see Supplementary Table 1). However, 15 patients (46.9%) in addition fulfilled diagnostic criteria for TS. In 10 (31.3%) of these, correct diagnosis of TS had been made before the onset of FND, while in four patients (12.5%), additional diagnosis of TS was made during presentation in our clinic (missing data: *n* = 1, 3.1%). In all cases, however, FTB represented the predominant symptom.

### MSMI-FTB

#### Functional motor symptoms (FMS)

While all patients reported “complex” FMS, only 19 patients (59.4%) presented with “simple” FMS. Altogether, 232 *different* FMS could be captured, of which 18 were classified as “simple” and 214 as “complex”. On average, each patient suffered from 1.4 (range: 0–6, median: 1) “simple” and 11.9 (range: 3–31, median: 10) “complex” FMS. In all but one patient (96.9%), FMS did not follow a rostro-caudal distribution. For details see Table 2.

**Table 2:** Most common functional motor and vocal symptoms: Type, localization, kind, frequency, and overlap with Jan Zimmermann's symptoms (*n* = 32).

**Symptom**	**Frequency *n* (%)**	**Symptoms presented by** ** Jan Zimmermann**
**Complex functional motor symptoms**		
Head movements Arm movements Trunk movements Hand movements Face movements (Eyes, Mouth, Lips, Gaze) Leg movements Altered gait	24 (75.0%) 20 (63.0%) 15 (47.0%) 12 (38.0%) 11 (34.0%) 10 (31.0%) 8 (25.0%)	Yes Yes Yes Yes Yes No Yes
**Obscene/socially inappropriate functional motor symptoms**		
Throwing objects and foodShowing middle fingerTouching othersHurting, hitting, biting oneselfHurting, hitting, kicking othersDestroying objectsHitting or kicking objects	20 (63.0%) 17 (53.0%) 15 (47.0%) 15 (47.0%) 12 (38.0%) 12 (38.0%) 12 (38.0%)	Yes Yes Yes Yes Yes Yes Yes
**Simple functional motor symptoms**		
Head movementsArm movementsTorso movementsHand movementsFace movements (Eyes, Mouth, Lips, Gaze)Leg movementsAltered gaitHead	24 (75.0%) 20 (63.0%) 15 (47.0%) 12 (38.0%) 11 (34.0%) 10 (31.0%) 8 (25.0%) 2 (6.0%)	Yes Yes Yes Yes Yes No Yes Yes
**Complex functional vocal symptoms**		
Arschloch (asshole)Fick dich (fuck you)Wichser (wanker)Fotze (cunt)Pommes (French fries)Nein (no)Halt die Fresse (shut up)	13 (40.6%) 12 (37.5%) 9 (28.1%) 8 (25.0%) 7 (21.9%) 7 (21.9%) 7 (21.9%)	Yes Yes Yes Yes Yes Yes Yes
**Thematic clusters of complex functional vocal symptoms**		
Fick^*^ (fuck^*^)Arsch^*^ (ass^*^)Right wing vocabularyFoodsDisagreementFotze^*^(cunt^*^)Wichser^*^ (wanker^*^)	20 (62.5%)15 (46.9%)14 (43.8%)13 (40.6%)12 (37.5%)11 (34.4%)9 (28.1%)	YesYesYesYesNoYesYes
**Animal sounds**	11 (34.4%)	Yes
**Simple functional vocal symptoms**		
Syllable “hm”Mouth clickingWhistlingScreaming	9 (28.1%)7 (21.9%)6 (18.8%)5 (15.6%)	YesYesYesNo

* Placeholder for different word combinations within the thematic clusters.

#### Functional vocal symptoms (FVS)

All patients presented with “complex” and 28 (88%) with “simple” FVS. The mean number of “simple” FVS was much lower (mean: 2.2, range, 0–6, median: 2) compared to that of “complex” FVS (mean: 18.8, range: 3–72, median: 15). Altogether, we identified 471 *different* FVS. In four patients (12.5%) the number of FVS was <10, in 15 (46.9%) between 10–30, in five (15.3%) between 31–60, and in eight (25.0%) between 100–200.

“Animal sounds” were identified in 11 patients (34.4%). Because of their high frequency and relative complexity, we classified them different from YGTSS as a separate category.

The spectrum of “complex” FVS ranged from single words to 10-word sentences with neutral, socially inappropriate, and offensive content, mainly in German, but partly also in English language. The two by far most often used “complex” FVS were the German swear words “Arschloch” (“asshole”) and “Fick dich” (“fuck you”). Since “complex” FVS showed a remarkable overlap, we clustered them depending on the topic as follows (in descending order): “fick/fuck”, other swear words, radical right-wing statements, food-related words, and statements expressing disagreement (for details see Table 2).

More than half of patients (*n* = 17, 53.1%) reported a change in their voice pitch (low: *n* = 9, 28.1%, high: *n* = 3, 9.4%, fluctuating pitches: *n* = 5, 15.6%) only occurring when pronouncing FVS.

### Premonitory sensations and suppressibility

Twenty-seven patients (84.4%) reported experiencing a premonitory sensation prior to the occurrence of FTB, in most patients located at the body part where FTB occurred (*n* = 14, 43.8%). Premonitory sensations were reported to last on average 66.5 s (*SD*: 166 s, range: 1 s-1 h, median: 3 s). Fifteen patients (46.9%) gave descriptions such as having “a stone collar around ones neck”, “dam inside the stomach”, or “trembling inside the head”. Twenty-seven patients (84.4%) reported being able to voluntarily suppress FTB on average for 71 min (*SD*: 128 min, range: 1 s-8 h, median: 15 min). For details see Table 1.

### Distractibility, aggravating, triggering, and improving factors

In 31 patients (97%) symptoms were distractible. Twenty-eight patients (87.5%) reported specific aggravating factors such as stress (*n* = 16, 50%), presence of other people (*n* = 13, 40.6%), anger (*n* = 3, 9.4%), tiredness (*n* = 3, 9.4%), expectations from others (*n* = 2, 6.3%), and others (*n* = 6, 18.8%) (multiple answers possible).

Nineteen patients (65.6%) reported specific triggering factors with symptom provocation by certain words, sentences, or sounds (e.g., shouting “beep beep” following a phone ring; *n* = 9, 28.1%), by specific characteristics of people (e.g., shouting “fette Kuh” (“fat cow”) when seeing an obese woman; *n* = 8, 25%), and by police officers [e.g., shouting “Bombe” (“bomb”) or “Bullenfotze” (“cop cunt”); *n*=5, 15.6%]. However, the most often reported triggering factor (*n* = 14, 43.8%) were certain relatives (parents: *n* = 13, 40.6%, partners: *n* = 4, 12.5%, siblings: *n* = 2, 6.3%, close friends: *n* = 2, 6.3%, and own child: *n* = 1, 3.1%, multiple answers possible) causing socially inappropriate behaviors and “complex” FVS with obscene words/phrases and socially inappropriate and offensive content.

All patients reported factors resulting in a symptom improvement and 25 (78%) even a complete remission for hours when concentrating (*n* = 14, 43.8%), relaxing (*n* = 10, 31.3%), practicing sports (*n* = 8, 25.0%), using the mobile phone or gaming (*n* = 7, 21.9%), in the presence of others (*n* = 6, 18.8%) or pets (*n* = 6, 18.8%), being alone (*n* = 3, 9.4%), and miscellaneous factors (*n* = 5, 15.6%, multiple answers possible).

### Injuries due to functional symptoms

Fifteen patients (46.9%) reported accidental self-injuries, but in no case, medical treatment was necessary. Nevertheless, 17 patients (53.1%) undertook precautionary measures to prevent possible injuries; nine (28.1%) avoided potentially dangerous objects such as knives; six (18.8%) used protective tools like cushions; three (9.4%) instructed family/friends to remove specific objects; two (6.3%) used plastic dishes; and four (12.5%) indicated others.

### Additional psychiatric and somatic diagnoses and symptoms

In all but two patients (93.8%), further psychiatric symptoms were found (mean: 4.3, range: 0–9, median: 4): abnormalities in social behavior such as difficulty fitting into social groups and problems making friends (*n* = 26, 81.3%), obsessive-compulsive behavior (OCB) (*n* = 15, 46.9%), anxiety (*n* = 13, 40.6%), depression (*n* = 10, 31.3%), sleeping problems (*n* = 8, 25.0%), autism spectrum disorder (ASD) (*n* = 5, 15.6%), personality disorders (*n* = 6, 18.8%, among them *n* = 5 (15.6%) of emotionally unstable type and *n* = 1 (3.1%) unspecified), suicidal ideation (*n* = 5, 15.6%), pre-existing FTB (*n* = 2, 6.3%), pre-diagnosed intellectual disability and comorbid conduct disorder (*n* = 3, 9.4%), and post-traumatic stress disorder (PTSD) (*n* = 2, 6.3%). Fifteen patients (46.9%) were exposed to bullying at some point in life.

Twenty-four patients (75%) reported somatic diseases (mean: 2.4, range: 0–12, median: 2): pre- or perinatal complications (*n* = 10, 31.3%), past invasive surgery (*n* = 9, 28.1%), allergies, headache, developmental delay, and physical disability requiring use of wheelchair or rolling walker (*n* = 3, 3.9%, each).

Disregarding social media induced FTB, 13 patients (40.6%) were considered severely mentally ill, four (12.5%) severely physically ill, eight (25.0%) as both, and seven (21.7%, all females) as neither.

### Psychological stressors, unconscious intrapsychic conflicts, and structural deficits

In all patients, unconscious intrapsychic conflicts (*n* = 11, 34.4%), structural deficits (*n* = 12, 65.6%) or both (*n* = 9, 28.1%) were found. Fourteen patients (43.8%) exhibited relevant autonomy-dependency-conflicts. We found a clear relationship between the presence of intrapsychic conflicts and structural deficits, respectively, and comorbid psychiatric symptoms with highest number of psychiatric symptoms in patients with intrapsychic conflicts *and* structural deficits (mean: 5), followed by those with structural deficits only (mean: 4.3) and with intrapsychic conflicts only (mean: 2.5). For details see Supplementary Table 2.

In 22 patients (68.8%), timely-related psychological stressors were identified: substantial conflicts in family, partnership, at school/work (*n* = 9, 28.1%), considerable life changes such as parents separation, change of class/school, moving out or back into the parental home, moving out of close siblings, or occupational disability (*n* = 8, 25.0%), and confrontation with exceptional life events such as impending/recent surgery and sudden hard lockdown due to the pandemic (*n* = 5, 15.6%). In six of these patients, in addition, considerable structural deficits and comorbidities (personality disorders, PTSD, and pre-existing FTB) were found. In the remaining 16 patients, timely-related psychological stressors reactivated unconscious intra-psychic conflicts.

Finally, in all of those patients (*n* = 19, 59.4%) still closely integrated in the family structure, dysfunctional dynamics between family members became apparent.

### Maintaining factors

In all but one patient (96.9%), maintaining factors could be identified (multiple answers possible) such as granting of special privileges at home/school/work (*n* = 19, 61.3%, e.g., permission to leave the classroom whenever wanted, to write exams in separate rooms without supervision, relief from everyday household duties or more difficult duties at work); receipt of special attention from parents/friends/partners with more loving and caring interactions, increased social recognition at school, or increased attention on social media (*n* = 18, 58.1%); self-experienced improvement of social interactions due to MSMI-FTB (*n* = 4, 12.9%); and feeling sensations of relief after performing FTB (*n* = 4, 12.9%, e.g., “I feel better/more relaxed afterwards;” three of these patients had severe structural deficits as described above).

Eleven patients (34.4%) reported displaying FTB on social media as tics/TS; another three patients (9.4%) each stated that they would like to do so, but parents would not allow or they would not be brave enough to do so.

### Social media use and overlap with “Gewitter im Kopf”

All patients confirmed having watched “Gewitter im Kopf” and 30 (93.8%) stated using YouTube regularly. In all patients, onset of FTB was after the YouTube channel was launched and in 29 patients (90.6%) after having watched “Gewitter im Kopf” (missing data: *n* = 3, 9.4%).

Based on a detailed analysis comparing patients' symptoms to those of the channel-host, there was an obvious overlap with respect to “complex” movements and vocalizations, inappropriate behaviors (*n* = 32, 100%, each), change in voice pitch when pronouncing FVS (*n* = 17, 53.1%), and giving FTB an old-fashioned German name (e.g., Günter, Uwe, Hildegard or Helga) similar to the channel-host, who named his disease “Gisela” (*n* = 17, 53.1%). For details see Table 2.

### Follow-up after diagnosis of social-media induced FTB

At follow-up (mean: 4.8 months, range: 6 days–19 months), 17 patients (53.1%) reported an improvement of FTB on average of 74.3% (range: 15–99%, median: 80%). Of these, in 4 (12.5%), symptoms improved without treatment, while 13 patients (40.6%) had received treatment, which consisted in 9 (28.1%) of these of not specified psychotherapy and in 4 (12.5%) of other not specified treatments. In four patients (12.5%) symptoms markedly improved immediately after the diagnosis had been made. In the majority, symptoms improved slower after 1–4 days (*n* = 2, 6.3%), 2 weeks (*n* = 1, 3.1%), and 2–6 months (*n* = 4, 12.5%, no data available: *n* = 6, 18.8%). Twenty-five patients (78.1%) accepted the diagnosis, but five (15.6%) still insisted on suffering from TS (no information: *n* = 2, for details see Table 3). Altogether, 23 patients (65.6%) sought treatment for their FTB.

**Table 3:** Reactions of patients (*n* = 32) and parents (*n* = 16) after having received the diagnosis of MSMI-FTB.

**Reactions**	**Patients *n* (%)**	**Parents *n* (%)**
Relief	12 (34.4)	14 (40.6)
Disappointment	6 (18.8)	3 (9.4)
Uncertainty	5 (15.6)	3 (9.4)
Anger	4 (12.5)	1 (3.1)
Indifference	1 (3.1)	0 (0)
Others	3 (12.5)	0 (0)
No information	1 (3.1)	11 (34.4)

MSMI, Mass social media-induced illness; FTB, functional Tourette-like behavior.

### Comparisons of subgroups

#### Males versus females

No significant sex differences were found (for details see Table 1).

#### Patients with MSMI-FTB with and without TS

When comparing tics in patients with comorbid TS (*n* = 15) and those without TS (*n* = 17), in the FTB only group we found (i) a significantly higher rate of abrupt onset (with a large effect) (*p*_*c*_ < 0.001, *V* = 0.88), (ii) a significantly higher rate of constant increase of symptoms (with a large effect) (*p*_*c*_ < 0.001, *V* = 0.76), and (iii) a significantly lower rate of OCB (with a large effect) (*p*_*c*_= 0.025, *V* = 0.62) (for details see Table 4).

**Table 4:** Patients with MSMI-FTB with comorbid TS (*n* = 15) compared to those without comorbid TS (*n* = 17).

	**FTB + TS (*n* = 15)**	**FTB (*n* = 17)**	***p* ^b^ ^,^ ^d^**	***p* *_c_* ^c^ ^,^ ^d^**
**Sex (male/female)**	9/6	7/10	0.480	1
**Age (year, mean+/-** ***SD*** **)**				
at evaluation at onset of tics at onset of FTB	20.7 +/- 12.9 7.3 +/- 2.9 19.8 +/- 13.0	19.6 +/- 9.4 NA 18.6 +/- 9.3	0.787 NA 0.762	1 NA 1
**Course of symptoms**				
Abrupt onset (*n*, %) Symptom fluctuations (*n*, %) Constant increase of symptoms (*n*, %)	0 (0%)^a^ 15 (100%)^a^ 1 (6.7%)^a^	15 (88.2%) 12 (70.6%) 14 (82.4%)	**<0.001** **0.046** ** <0.001**	**<0.001** 1 ** <0.001**
**Clinical characteristics of symptoms**				
Premonitory sensation (*n*, %) Suppressibility (*n*, %) Rostro-caudal distribution (*n*, %) Distractibility (*n*, %) Temporary situational remission (*n*, %)	11 (73.3%)^a^ 8 (53.3%)^a^ 0 (0%) 14 (93%) 10 (67%)	16 (94.1%) 16 (94.1%) 1 (6 %) 17 (100%) 15 (88%)	0.161 **0.013** 1 0.488 0.210	1 0.325 1 1 1
***n*** **of FTB (mean** **+/-** ***SD*****)**				
Simple motor symptoms Complex motor symptoms Simple vocal symptoms Complex vocal symptoms Obscene and socially inappropriate symptoms	0.7 +/- 1.1 12.5 +/- 9.2 2.3 +/- 1.9 21.9 +/- 16.7 15 (100%)	1.9 +/- 1.7 11.5 +/- 6.7 2.1 +/- 1.2 16.1 +/- 10.5 16 (94.1%)	**0.027** 0.727 0.715 0.238 1	1 1 1 1 1
**Comorbidities**				
ADHD (*n*, %) OCB (*n*, %) Anxiety (*n*, %) Depression (*n*, %) ASD (*n*, %)	0 (0%) 12 (80.0%) 6 (40.0%) 5 (33.3%) 4 (26.7%)	3 (17.7%) 3 (17.7%) 7 (41.2%) 5 (29.4%) 1 (5.9%)	0.229 **0.001** 1 1 0.161	1 **0.025** 1 1 NA
**Further psychiatric disorders/symptoms**				
Abnormalities in social behavior Personality disorder Sleeping problems Suicidal ideation Exposure to bullying	13 (86.7%) 4 (26.7%) 5 (33.3%) 3 (20.0%) 7 (46.7%)	13 (76.5 %) 2 (11.8 %) 3 (17.7 %) 2 (11.8 %) 8 (47.1 %)	0.659 0.383 0.424 0.645 1	1 1 1 1 1

MSMI, Mass social media-induced illness; TS, Tourette syndrome; FTB, functional Tourette-like behavior; SD, standard deviation; NA, not applicable; ADHD, attention deficit/hyperactivity disorder; OCB, obsessive-compulsive behavior; ASD, autism spectrum disorder.

a Refers to tics, all other numbers refer to FTB.

b Uncorrected p-values.

c Corrected p-values according to Bonferroni.

d t-tests were performed for interval-scaled dependent variables and Fisher's exact tests for dichotomous dependent variables; Significant results are shown in bold.

## Discussion

In this study, we present for the first time an in-depth characterization of a large cohort of patients with MSMI-FTB. Our main findings are: (i) we found a large symptom overlap within our sample, but also with symptoms presented by the host of the German YouTube channel “Gewitter im Kopf” justifying our recently suggested concept of a new type of MSI that—in contrast to recent outbreaks—spreads solely *via* social media and is induced by a “virtual” index case (1); (ii) in all patients, timely-related psychological stressors, unconscious intrapsychic conflicts, and/or structural deficits could be identified predisposing for contagion with MSMI-FTB; (iii) in nearly half of our patients, pre-existing TS was diagnosed suggesting tics as another independent predisposing factor for MSMI-FTB; (iv) in most patients, abnormalities in social behavior and several further psychiatric symptoms were diagnosed; (v) in contrast to other recent reports (6–8, 10), half of our patients were male; however, there were no clinical differences between males and females; and (vi) preliminary follow-up data show that the course of MSMI-FTB spans widely from spontaneous complete remission within days to no improvement after months.

Only recently, an increasing, but still small number of reports has been published describing mainly young people with FTB. When comparing main characteristics of our sample with previous reports (5–7, 11), a number of similarities, but also relevant differences could be identified. Similarities included (i) mainly rapid onset of symptoms (6–8, 10); (ii) mainly rapidly progressive course (6, 7, 11, 12); (iii) positive history of exposure to social media content related to FTB (5–7, 9, 11); (iv) triggering factors provoking certain symptoms (12); (v) history of coexisting medically unexplained symptoms including other FTB (9); (vi) very similar pattern of movements and vocalizations across different centers, countries, and continents with predominantly “complex” movements involving the trunk, upper extremities, and head as well as “complex” vocalizations with swear words and insults (6, 7, 9, 11, 12). This observation is in line with our hypothesis that spread of MSMI induces within a short time period “secondary virtual” index cases resulting in a global—and no longer locally restricted—outbreak (1); and (vii) misdiagnosis of tic disorder/TS, before patients are seen in specialized TS outpatient clinics (11).

Different from available reports, we describe for the first time a small number of older patients (*n* = 4, three females, aged 30, 49, 51, and 53 years) affected with MSMI-FTB. However, in line with other reports (5, 7, 9, 11), the majority of our patients were adolescents or young adults (range: 11–23, median age: 18 years). In all patients over age 30, obvious structural deficits were found and in three of them, in addition, clinically relevant comorbidities such as abnormalities in social behavior (*n* = 3), TS, personality disorders, and PTSD (*n* = 2, each). This higher burden of psychopathologies may explain worse prognosis in adults compared to adolescents as reported recently by Howlett et al. (33).

In contrast to recent studies from Canada, United Kingdom, and the United States reporting a female preponderance of about 9:1 (5–7, 9), in our sample males and females were equally distributed. Noteworthy, in another German sample (*n* = 13), an even higher male to female ratio (1.6:1) was found (11). In line with our concept of MSMI (1), we believe that different sex ratios are related to the fact that in Germany with Jan Zimmermann (15) a male person acts as the “virtual” index case, while in English speaking countries the most influential person (“thistrippyhippie”) (17) is a female. Based on research by Bartholomew et al. (2) it is well-known that spread in MSI outbreaks is triggered by emotional arousal and identification. Since emotional contagion also spreads *via* social media (34, 35) channel-hosts' sex may have an impact on sex ratios. Accordingly, most of our (male) patients reported finding the channel-host likable—at least in the beginning.

Our detailed clinical characterizations enables us to add the following aspects to the existing literature: (i) numbers of complex movements and vocalizations were nine times greater than of “simple” symptoms, and of vocalizations one and a half times greater than of movements; (ii) premonitory sensations and suppressibility are often described, but largely differ from typical reports in patients with tics. When comparing our results to reports in TS, premonitory urges in TS are described as a feeling of pressure or tension located in the same body area as the corresponding tic lasting only milliseconds and thus are less complex, shorter, with less variants, and more circumscribed (36, 37). In TS, patients report to be able to suppress their tics on average for a few minutes and thus much shorter and with much less contingency (36, 37); (iii) aggravating, triggering, and improving factors are characteristic features for MSMI-FTB. Although patients with TS also report environmental factors with stress, anxiety and fatigue being the most common factors that may transiently exacerbate tics (38), descriptions clearly differ from those in patients with MSMI-FTB. For example, in MSMI-FTB, on average a much larger number of influencing factors is reported, symptoms often increase in the presence of other people, while tics in TS usually decrease when other people are around (39), and most patients report very specific or even rather peculiar triggers such as the presence of certain people e.g., teachers who are disliked, or calls for unloved obligations. Furthermore, a complete remission of symptoms for hours or even days or weeks as often described by patients with MSMI-FTB is rather unusual in patients with TS. However, similar to patients with TS, patients with MSMI-FTB most often report an improvement of symptoms while relaxing and concentrating (39); (iv) distractibility is a well-known phenomenon in FND that was also often seen in our sample of MSMI-FTB, while it is only partially present in patients with TS (40); (v) maintaining factors were found in nearly all patients and seem to play an important role in symptom persistence; and (vi) minor injuries due to functional symptoms are common often inducing disproportionate precautions.

With respect to obscene/socially inappropriate functional symptoms, it is well-known that coprophenomena are complex tics that occur only in a small percentage of patients with TS (41). In addition, coprolalia in TS is usually characterized by only a small number of single words (and not countless and very complex utterances) that are often in addition masked by the pronunciation of only the first letters (41, 42). Relation to the context might be another factor that may help to differentiate coprophenomena in TS from obscene/socially inappropriate functional symptoms in FTB. However, a clear distinction of obscene/socially inappropriate functional symptoms from non-obscene socially inappropriate behavior (NOSI) can be difficult and raises the question how to interpret NOSI in TS in general (43). Currently, there is increasing evidence that in TS a functional overlay is much more common than previously thought (unpublished data).

Since first cases with MSMI-FTB occurred about 1 year (first onset in first patient: 2/2019, first presentation of first patient in our clinic: 5/2019) *before* the pandemic (first COVID-19 case in Germany: 1/2020), it can be excluded that the pandemic is the primary cause of this outbreak. However, it may have fueled spread due to increased social media use, increased anxiety, and loss of compensatory factors such as reduced personal contacts, hobbies, and daily activities (1), since during the pandemic a general increase in FND has been observed (12).

In line with recent reports (6, 7), we found anxiety and depressive symptoms in a substantial number of patients. However, apart from that, we detected a broad spectrum of somatic as well as further psychiatric diagnoses and symptoms including OCB, sleeping problems, and ASD in all but two patients, comorbid TS in nearly half of patients, abnormalities in social behavior in more than four-fifth, structural deficits in two-third, experience with bullying in nearly half of patients, and timely-related psychological stressors and underlying unconscious intrapsychic conflicts in about two-third of patients. Thus, it can be hypothesized that various factors predispose for contagion with MSMI-FTB that may be enhanced by dysfunctional family relations. From a psychodynamic perspective, it can be assumed that timely-related psychological stressors reactivated unconscious inner psychological conflicts and thus enabled destabilization of an already fragile psychological balance in individuals with pre-existing structural deficits (30). With respect to treatment, we recommend to also take these underlying psychopathological pattern as well as dysfunctional family relations into consideration. Since only one-third of our patients and 40% of parents felt relieved after being informed about the diagnosis of MSMI-FTB, more general treatment recommendations for FND may be useful (44) including an empathic and reassuring basic attitude.

Of fundamental importance is the question why the first ever documented outbreak of MSMI presented with FTB. Following the LeRoy outbreak in 2011 (45), this is the second outbreak described presenting with FTB. Specific symptomatology of both FND in general (46) and the “motor variant” of MSI (47–49) is known to be cultural-bound and closely related to social demands. The majority of patients affected with MSMI-FTB are adolescents and young adults. This suggests that the period of identity formation characterized by questioning oneself, seeking new identities, and trying out new roles (50) plays a part. Since a substantial number of patients exhibits autonomy-dependence conflicts, it can be speculated that social media influencers serve as alternative role templates resulting in functional symptoms primarily characterized by taboo words, insults, offensive comments, socially inappropriate behaviors, and transgressive attitudes. Remarkably—and in line with recent reports (10)—a small number of our patients in parallel were engaged with issues of their own gender identity.

The following limitations have to be addressed: (i) a selection bias cannot be excluded, since it can be assumed that more severely affected patients presented in our center; (ii) some answers were conflicting and unreliable and might be biased due to parental supervision during the interview, unawareness of subconscious conflicts within the family or complex psychodynamic situations; (iii) in some of the adult patients only self-reports were used which might have caused symptoms under- or overestimation (iv) in two patients, psychological interviews were performed with the parents only and not the patients. One of these interviews was done only *via* phone; (v) although this is the largest sample of MSMI-FTB, the sample size is still relatively small; (vi) although data were collected prospectively using a semi-structured interview, accuracy of statements regarding psychopathological background including intrapsychic conflicts and structural integration can be further increased by using a standardized questionnaire; and (vii) another subject of future research should also be the investigation of the role of education and occupational status in the development of FTB.

## Data availability statement

The original contributions presented in the study are included in the article/Supplementary material, further inquiries can be directed to the corresponding author.

## Ethics statement

The study was reviewed and approved by Local Ethics Committee at Hannover Medical School (No. 8995_BO_S_2020). Written informed consent to participate in this study was provided by the participants and their legal guardian/next of kin.

## Author contributions

CF contributed to conception and design of the study, organization of the database, collection, analysis and interpretation of data, and wrote the first draft of the manuscript. NS contributed to data analysis and interpretation. AP contributed to the conception of the study, organization of the database, and data analysis. MH contributed to the statistical analysis of the study. LL contributed to the organization of the database and the collection of the data. CW contributed to the conception and design of the study. KM-V contributed to conception, design of the study and collection, and analysis and interpretation of data. All authors contributed to manuscript revision, read, and approved the submitted version.

## Conflict of interest

Author KM-V has received financial or material research support from EU (FP7-HEALTH-2011 No. 278367, FP7-PEOPLE-2012-ITN No. 316978) DFG: GZ MU 1527/3-1 and GZ MU 1527/3-2, BMBF: 01KG1421, National Institute of Mental Health (NIMH), Tourette Gesellschaft Deutschland e.V. Else-Kröner-Fresenius-Stiftung, GW pharmaceuticals, Almirall Hermal GmbH, Abide Therapeutics, and Therapix Biosiences. She has received consultant's honoraria from Abide Therapeutics, Boehringer Ingelheim International GmbH, Bionorica Ethics GmbH, CannaMedical Pharma GmbH, Canopy Grouth, Columbia Care, CTC Communications Corp., Demecan, Eurox Deutschland GmbH, Global Praxis Group Limited, IMC Germany, Lundbeck, Sanity Group, Stadapharm GmbH, Synendos Therapeutics AG, and Tilray. She is an advisory/scientific board member for CannaMedical Pharma GmbH, Bionorica Ethics GmbH, CannaXan GmbH, Canopy Growth, Columbia Care, IMC Germany, Leafly Deutschland GmbH, Sanity Group, Syqe Medical Ltd., Therapix Biosciences Ltd., and Wayland Group. She has received speaker's fees from Aphria Deutschland GmbH, Almirall, Cogitando GmbH, Emalex, Eurox Deutschland GmbH, Ever pharma GmbH, Meinhardt Congress GmbH, PR Berater, Spectrum Therapeutics GmbH, Takeda GmbH, Tilray, and Wayland Group. She has received royalties from Deutsches Ärzteblatt, Der Neurologe und Psychiater, Elsevier, Medizinisch Wissenschaftliche Verlagsgesellschaft Berlin, and Kohlhammer. The remaining authors declare that the research was conducted in the absence of any commercial or financial relationships that could be construed as a potential conflict of interest.

## Correction note

A correction has been made to this article. Details can be found at: 10.3389/fpsyt.2025.1687567.

## Publisher's note

All claims expressed in this article are solely those of the authors and do not necessarily represent those of their affiliated organizations, or those of the publisher, the editors and the reviewers. Any product that may be evaluated in this article, or claim that may be made by its manufacturer, is not guaranteed or endorsed by the publisher.

## Footnotes

^1^ https://www.youtube.com/channel/UCh2Nc3OwjSwuXrUdFNXqFbQ

^2^ https://www.tiktok.com/@thistrippyhippie

^3^ https://www.youtube.com/channel/UCJHvN0zYgO2ZLjePERRLjKQ

^4^ https://www.instagram.com/stine_sara_tourette/?hl=de

^5^ https://socialblade.com/youtube/channel/UCh2Nc3OwjSwuXrUdFNXqFbQ

^6^ https://www.youtube.com/watch?v=F-3YEoIs-vs

^13^ https://www.youtube.com/watch?v=BtqZ3HsoNeY

## Abbreviations

ADHD: attention deficit/hyperactivity disorder
ASD: autism spectrum disorder
DSM-5: Diagnostic and Statistical Manual of Mental Disorders
FMS: Functional motor symptoms
FND: functional neurological disorders
FTB: functional “Tourette-like” behavior
FVS: Functional vocal symptoms
MSI: mass sociogenic illness
OCB: obsessive-compulsive behavior
PTSD: post-traumatic stress disorder
TS, Tourette syndrome, MSMI: mass social media-induced illness
YGTSS: Yale Global Tic Severity Scale.
