# Correction: Mass social media-induced illness presenting with Tourette-like behavior

**DOI:** 10.3389/fpsyt.2025.1687567

**Published:** 2025-09-15

**Authors:** Carolin Fremer, Natalia Szejko, Anna Pisarenko, Martina Haas, Luise Laudenbach, Claudia Wegener, Kirsten R. Müller-Vahl

**Affiliations:** ^1^ Clinic of Psychiatry, Social Psychiatry and Psychotherapy, Hannover Medical School, Hanover, Germany; ^2^ Department of Neurology, Medical University of Warsaw, Warsaw, Poland; ^3^ Department of Bioethics, Medical University of Warsaw, Warsaw, Poland; ^4^ Department of Audiovisual Media Studies, Film University Babelsberg, Potsdam, Germany

**Keywords:** Tourette-like behavior, social media, mass sociogenic illness, functional movement disorders, mass social media-induced illness

The statistical parameters for “complex” FVS were incorrectly given as “(mean: 43, range: 3–200, median: 21.5)”.

A correction has been made to the section **Results**, *MSMI-FTB, Functional vocal symptoms (FVS)*, Paragraph 1:

“The mean number of “simple” FVS was much lower (mean: 2.2, range, 0-6, median: 2) compared to that of “complex” FVS (mean: 18.8, range: 3-72, median: 15).”

The original version of this article has been updated.

