# Supplementary material for: Mass social media-induced illness presenting with Tourette-like behavior
Source: Front Psychiatry. 2022 Sep 20;13:963769. doi: 10.3389/fpsyt.2022.963769 (PMC9530444; doi:10.3389/fpsyt.2022.963769)
Supplement: Supplementary file 1 [file Table_1.docx]

Supplementary Material

**Supplementary Table 1.** Type and reported effects of medication used for treatment of social media-induced functional Tourette-like behavior (FTB)

| **Medication** | ***n* of patients**  **(%)** | **Discontinued^a^ (*n*, %)** | **Duration^b^,**  **mean [months]** | **Max dose/d**  **(mean)** | **Beneficial effects^c^**  **(*n* initially/long-lasting,**  **mean %^d^)** | ***n* of side effects**  **(mean)** | **Reported side effects** |
| --- | --- | --- | --- | --- | --- | --- | --- |
| Aripiprazole | 8 (25%) | 7 (21.9%) | 5.4 | 8.7mg | 4/2 (26.3%) | 3 | apathy, speech delay, weight gain, fatigue, difficulty falling asleep, loss of appetite, dizziness, nausea, emotionally cold, abdominal cramps, "tic attacks”, stuffy nose |
| Tiapride | 6 (18.8%) | 6 (18.8%) | 8.0 | 287.5mg | 3/0 (18%) | 2 | fatigue |
| Risperidone | 5 (15.6%) | 4 (12.5%) | 1 | 0.83mg | 3/1 (43%) | 3 | difficulty falling asleep, headaches, flabby muscles, abdominal pain |
| Medical cannabis | 3 (9.4%) | 0 (0%) | 6.5 | 1.65g | 4/3 (45%) | 2 | cough, light fatigue |
| Lorazepam | 3 (9.4%) | 1 (3.1%) | MD | MD | MD | MD | MD |
| MPh | 2 (6.3%) | 1 (3.1%) | MD | MD | 0/0 (0%) | 2 | increase of FTB, abdominal pain |
| Dronabinol | 2 (6.3%) | 1 (3.1%) | 7.6 | MD | 0/0 (0%) | MD | MD |

FTB – functional Tourette-like behavior, Max – maximum, d – day, MD – missing data, MPh – Methylphenidate

^a^ before visit in our clinic

^b^ of treatment

^c^ on FTB, given in % of reduction of FTB

^d^ of overall improvement of FTB

**Supplementary Table 2.** Relationship between unconscious intrapsychic conflicts, structural deficits and comorbidities in patients with social media-induced functional Tourette-like behavior (FTB) (*n*=32)

|  | *n* of patients (%) | Comorbidities | | | | | | | | | |
| --- | --- | --- | --- | --- | --- | --- | --- | --- | --- | --- | --- |
|  |  | Total number,  mean (range, median) | ADHD,  *n* (%) | OCB,  *n* (%) | Anxiety,  *n* (%) | Depression,  *n* (%) | ASD,  *n* (%) | Abnormal social behaviour,  *n* (%) | Personality disorder,  *n* (%) | Sleeping problems,  *n* (%) | Suicidal ideation,  *n* (%) |
| Intrapsychic conflicts only | 11 (34.38%) | 2 (0-4, 2) | 1 (3.23%) | 2 (6.3%) | 2 (6.3%) | 0 (0%) | 0 (0%) | 5 (15.6%) | 0 (0%) | 1 (3.1%) | 0 (0%) |
| Structural deficits only | 12 (37.50%) | 4.3 (1-9, 4) | 1 (3.23%) | 5 (15.63%) | 4 (12.5%) | 3 (9.4%) | 3 (3.1%) | 10 (31.5%) | 6 (18.8%) | 3 (9.4%) | 1 (3.1%) |
| Co-existing intrapsychic conflicts and structural deficits | 9 (28.13%) | 5 (0-8, 5) | 1 (3.23%) | 5 (15.63%) | 5 (15.63%) | 7 (21.9%) | 2 (12.5%) | 8 (25%) | 0 (0%) | 3 (9.4%) | 4 (12.5%) |

FTB – functional tic-like behaviors, ADHD – attention deficit/hyperactivity disorder, OCB – obsessive compulsive behavior, ASD – autism spectrum disorder
